# Amazonian destruction, Bolsonaro and COVID-19: Neoliberalism unchained

**DOI:** 10.1177/0309816820971131

**Published:** 2021-06

**Authors:** Paul Stewart, Brian Garvey, Mauricio Torres, Thais Borges de Farias

**Affiliations:** Grenoble Ecole de Management, France; University of Strathclyde, UK; Federal University of Para, Brazil; Independent Journalist

**Keywords:** Covid-19, rainforest fires, neo-liberal authoritarianism, accumulation by dispossession, creative destruction, Lula

## Abstract

During the current pandemic, forest loss in 2020 has dwarfed the devastation of the previous year. The scale of environmental crimes and aggression towards indigenous peoples and people of African-descendent has been a characteristic of the Bolsonaro administration in the Amazon region. As cases of COVID-19 rise daily in remote areas of the Amazon, a recent study indicates that indigenous lands that aren’t formally demarcated are more vulnerable to intrusion and hence disease: indeed illegal loggers have emerged as a key vector of Covid-19 transmission in a region with Brazil’s lowest number of intensive care units. The weakening of environmental protection in the Amazon has been systematic and a feature of the Brazilian shift from neo-liberalism to neo-developmentalism which can be characterised politically as neo-liberal authoritarianism. If Covid-19 also is now becoming a metaphor for the poisonous spread of neo-liberal globalisation, plunder and land grabs in the Brazilian rainforest can be seen to represent the most egregious of many egregious cases on the ground zero of neo-liberalism unchained. With the rise of Bolsonaro, we can see that the previous conjuncture characterised by the hegemony of PT and Lula was the exception to Brazil’s long embrace of the caudillo going back to the 1930s. Even then, a look at the mechanism of Lula’s rule raises questions as to precisely what changed under Lula when it came to the state and the rule of big capital.

## Ecological and epidemiological crisis

The Amazon fires of 2019 accompanied the greatest single-year loss of Brazilian forest in a decade (*The Guardian* 2019). With the world in the grip of a pandemic, however, forest loss in 2020 has dwarfed the devastation of the previous year. In April 2020 alone, 529 sq km of forest was destroyed – an increase of 171% on April 2019 (TerraBrasil 2020). The facilitation of environmental crimes and aggression towards indigenous, agrarian reform and African-descendent communities has been a particular hallmark of the Bolsonaro administration in the Amazon region with distinct social implications.^1^ As cases of COVID-19 grow by the day in remote areas of the Amazon, a recent study indicates that indigenous lands that aren’t formally demarcated are more vulnerable to intrusion and hence disease, with illegal loggers emerging as a key vector of COVID-19 transmission in a region with Brazil’s lowest number of intensive care units. Of the 10,300 cases of COVID-19 confirmed among indigenous people living in the country by July, there have been an estimated 408 deaths, with 347 in the Amazon region (Globo 2020).

**Figure 1.**
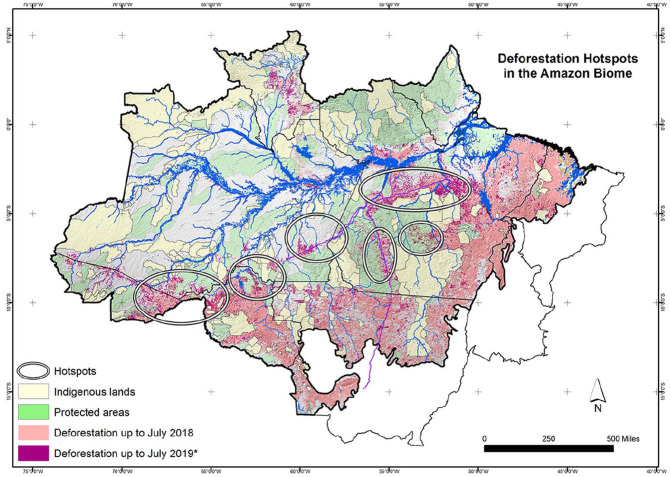
Deforestation hotspots in Amazon biome. Mauricio Torres.

The weakening of environmental protection in the Amazon has been systematic. Bolsonaro has challenged the Federal Justice order for the government to establish bases for environmental inspectors to restrict illegal logging and mining in hotspots of felling and burning (Ministério Público Federal 2020). These are areas in the Amazon where 60% of all deforestation occurs (Figure 1). With attention turned to the health crisis, the Minister of the Environment, Ricardo Salles, sacked the director of the federal environmental inspection agency, IBAMA, who had overseen a successful anti-mining operation on indigenous land in the interior of Pará and the Brazilian government reduced the budget for the environmental inspection agency IBAMA by 25% (Amazonia 2020). Many of the recent setbacks in Brazil’s environmental policy could be explained by a video that was released by court order on 22 May. The video shows a meeting between Bolsonaro and his ministers from a month before, in which environment minister Salles suggests the government take advantage of press attention being focused on the pandemic to relax regulations in the Amazon (*The Guardian* 2020).

The Brazilian Amazon may be on the eve of a catastrophe. COVID-19 could decimate indigenous communities, while the government response paves the way for profiteers to further degrade their lands and the forest. Bolsonaro’s legacy may be one of the highest national death tolls during the pandemic, and a point of no return for destruction of the Amazon

## Brazil: back to the future, forward to the past

More than a chilling depiction for the rapacious, ‘creative destruction’ of late capitalism in the era of neoliberalism, the burning of the Brazilian rainforest is part of the story of accumulation by dispossession (Harvey 2001) characterised by violent assaults on human and ecological resources worldwide. If COVID-19 also is now becoming a metaphor for the poisonous spread of neoliberal globalisation, plunder and land grabs in Brazilian rainforest could be seen to represent perhaps the most egregious of many egregious cases on the ground zero of neoliberalism unchained.

Today, in the 21st century, when writing about the social and political peculiarities of Bolsanaro’s authoritarian regime it would be easy to see its exclusionary and repressive signature as exceptional rather than a return, taking conjuncture, strategy and personnel into account, to the caudillo of Brazil since the 1930s (Quartim 1971). Indeed, with the rise of Bolsonaro and the social classes and political forces that propelled him to office, it is the previous conjuncture characterised by the hegemony of PT (Workers’ Party) and Lula, which can be seen to have been exceptional. Or perhaps not. Even then, a look at the mechanism of Lula’s rule raises questions as to precisely what changed under Lula in respect to state structure, political processes and the rule of big capital. While of course not in any sense comparable to the political project of incorporation–subordination introduced under Vargas’ *Estado Novo* (between 1937and 1941 social welfare legislation to contain class conflict including the 8-hour day, paid holidays and a minimum wage (Quartim 1971: 28)), nevertheless, PT’s rise to government never challenged the rule of capital and indeed deepened subservience to agribusiness (Oliveira 2009): the working class may have been in power but were never in control, the fate of social democratic governments everywhere today (Frankel 1997; Streeck 2017). The path from dictatorship to the liberal democratic ascendance of working-class political forces in 2003 is, therefore, crucial in any exploration of the current deepening of social subordination always central to the process of accumulation by dispossession.

## Background to Bolsonaro’s neoliberal authoritarianism

The military regime that took control following the CIA-backed overthrow of the leftist President Goulart oversaw the so-called Brazilian ‘economic miracle’ between 1964 and 1973 with the gross domestic product (GDP) growing annually by 11% between 1964 and 1973. Imports were crucial being dependent on oil but also international credit. Military rule ensured savage wage control and labour movement repression. In the period immediately following the 1973 oil crisis, Brazil’s petroleum-based imports increased significantly while the state continued to subsidise the involvement of international companies in massive infrastructure projects such as the TransAmazonia highway (Ianni 1979). In 1974, Brazil borrowed more money than it had in the previous 150 years. Following massive interest rate hikes in 1979 after the second oil crisis and in 1982, the country experienced the greatest debt default in post-war history. Enter the IMF (International Monetary Fund)!

Unsurprisingly, as social programmes were further trashed, the poorest 20% of the population saw their share of income drop from 3.9% in 1960 to 2.8% in the early 1980s, driving protests around social, labour and land reform from which would emerge CUT, PT and MST (Movimento dos Trabalhadores Sem Terra – Landless Peoples’ Movement). Despite the power and ingenuity of these movements, the new democratic opening in 1985, which preceded the formulation of a new constitution in 1988, failed to guarantee economic rights.

IMF and World Bank-driven structural adjustment programmes saw deregulation of state owned enterprises, the lifting of import tariffs and the end of credit support for the rural poor. As migration to the cities gathered pace by 2001 the favelas became home to 15 million unemployed people; the country saw a massive increase in work precarity and the neoliberal model was discredited to such an extent that space opened up for political change.

## From neoliberalism to neodevelopmentalism

Brazil’s Workers’ Party was central to articulating the mobilisation of a broad range of social and labour movements throughout the 1980s and early 2000s, but it would become apparent that this class base of workers and the dispossessed was not in itself sufficient to change the class character of the state. By the time of its successful election to power in 2002, PT’s centre of gravity had already shifted demonstrably from its anti-capitalist origins (Antunes 2019), while the party did not have parliamentary majorities sufficient to bring any change without deal-making. Furthermore, the loyalty of social movement leaderships and the co-option of CUT trade unionists into power left the union base poorly organised. The more radical trade unionists were already turning their backs on Lula. Perhaps this was not surprising since as Antunes (2019) has argued, PT had ‘converted itself into a party ‘for all’, ‘able to take power’ without challenging the status quo’ (p. 3).

This is in no sense to diminish the important social and economic character of the gains under PT, including the real minimum wage enhancements, the construction of and broader access to public universities, and the oft-cited 2004 *Programa bolsa famolia*^2^ (PBF); rather it is to argue that it is impossible to make sense of the relative ease with which Bolsonaro’s *ersatz* dictatorship rose to power without identifying the relatively fragile impact of state transformation under Lula.^3^ Many hoped that the Workers’ Party 2003 electoral victory would be the first nail in the coffin of neoliberalism, a kick in the backside of the neoliberal consensus that had been dominant since the 1980s. What PT did, however, was to introduce a modest degree of *dirigisme* into industry together with meek social and public policy reforms.

While aiming to reduce massive social inequalities, the perception was that this could be achieved via a push towards primary commodity production and exports. Specifically, an increasing reliance on China’s purchase of foodstuffs, agricultural produce more generally, and inevitably metals/ores (see inter alia, Garvey et al. 2019; Wilkinson and Wesz Jr. 2013) and incentivising domestic consumption (e.g. white goods and cars). Foreign direct investment (FDI) in minerals, agriculture and farm animals saw a huge rise: US$2.4 billion in 2000 to US$13.1 billion by 2007, while the conditional cash payments of the Bolsa Familia programme (PBF) increased household purchasing power. It was the social and political motivations and mobilisations which gave the PBF its significance rather than its class-shifting character, which was limited, and dependant on the commodities boom, the end of which heralded a new assault on the working class. Making sense of the import of cash transfer payments under the PBF is important since it illustrates both the context and time of this provision. It was a product of class conciliation, the modus operandi for *soi-disant* gains leveraged by social democratic parties in the current neoliberal era: enticements-cum-inducements sold as win–win outcomes even though, in the case of the PBF, the ability to deliver for labour was inextricably tied to the fortunes, literally, of finance and multinational capital. The privatisations required by capital in exchange for support for PTs limited social programmes were very significant. Not long after the Workers’ Party’s demise, Petras’ (2016) assessment was bleak:

“If we examine Brazilian merger and acquisitions activity and investment bank revenues, one sees a close correlation with the rise and fall of the PT regime. In other words, when the bankers, speculators and monopolists flourished under the PT policies, they supported the government of Lula and Dilma”.Moreover,

“With the recession fully underway, the business and banking elite demanded large-scale, long-term cuts in public expenditures, slashing budgets for the poor, education, health, housing and pensions, severe wage reduction and a sharp limit on consumer credit. At the same time they pushed through the privatization of the multi-billion dollar petroleum industry [Petrobras] and related state industries [. . .] and whatever else among Brazil’s public jewels could compensate for their drop in investment bank revenues and management fees for M&As”. (Petras 2016)

Thus, the limited character of social and political change under PT played no small part in the means and manner of Bolsonaro’s triumph. While we write ‘limited’ they were nevertheless important, but one problem was that they were insufficiently deep and extensive to weather the storm of neoliberal attack. Large sections of the working class who had benefitted directly from PT saw some of their gains being whittled away; recession led to unpopular austerity measures by Dilma; rising costs and unemployment. It is fair to say a large portion of the working class, disillusioned with the spending on mega projects such as the Olympics and World Cup, joined protests that (abetted by the conservative mainstream and social media campaigns) eventually turned on the government and PT, especially with the so-called operation ‘carwash’ corruption scandal. The Right was able to present PT as criminal, corrupt and this played into the hands of Bolosnaro’s populism. The latter successfully constructed an electoral bloc around a reactionary politics that included the capture of ‘anti-corruption’ sentiment, moral virtue (e.g. anti LGBT attacks) and ‘social order’ that tied up a huge percentage of the evangelical vote, which has huge influence across society, including the poor, urban peripheries and favelas.

Critical aspects of Petras’ argument accepted, nevertheless, the depth of the assault upon the working class, including labour and migrant workers, would not have been so extensive, rapacious, and destructive of human and physical resources had the gains made by subordinate social forces after 2003 not been perceived to have been so significant in material terms. That is to say that the nature of capital’s offensive under Bolsonoro’s regime highlights the fact that the limited gains made, in historical terms, were highly threatening to capital. Nor would the material aggression towards the environment, the killings of social movement opponents of capital’s new cycle of accumulation by dispossession, been so insistent had the limited *cultural–ideological* gains, and wider social promise of a socially progressive government not been so threatening to the neoliberals. The PSDB (Brazilian Social Democracy Party, a centrist formation) would not tolerate a further 4 years had PT’s Dilma won the election. That would have left the Right out of power for well over a decade. Members of other parties who would have voted with PT in exchange for favours turned to PSDB and this led to Dilma’s impeachment. Let’s just call it a coup. The solution hit upon, favoured by some though not necessarily all neoliberals was Bolsonaro, the latest in a long line of caudillos, whose legacies provide worrying signs for the future.

Yet, the Bolsonaro project is fraught with insecurities. Like his mentor in the north,^4^ his displays of narcissistic insecurity and contempt for opposition of any kind both obscures his broader agenda while masking the terror of his modus operandi. His project represents an attempt, so far with some success, especially as regards environmental theft and destruction, to push back labour by the neoliberal state unchained. It may not be so surprising that the dominant social classes in a country characterised by the *longue durée* of class incorporation–subordination, typically ruled by neofascist and military dictatorship for most of the 20th century, should have returned to the form of political class rule with which they were more comfortable following the onset of the 2008 crisis. Indeed, it is worth noting that under Bolsonaro, there are more military ministers today than during the period of the dictatorship (1964–1985), while the powerful rural caucus of land and business owners has increased its number of parliamentary seats.

Bolsonaro has all but buried the class conciliation project of the preceding Workers’ Party and the agency of class is made to disappear by a manipulative media-legal-political-ecclesiastical system. With a nostalgia towards the military counterrevolution, which ‘straightened’ society, capital’s ‘endemic, cumulative, chronic and permanent’ structural crisis (Mészáros 2011: 11–12) is being masked by moral preoccupations whereby migrants, women, reds and gay people are blamed for the supposed social deterioration. Those who have not subscribed to the market-liberal/social-autocratic reality of daily exclusion and micro-aggression against social and political minorities have faced sustained denigration.

Bolsonaro’s callous disregard for COVID-19 infections that have escalated in Brazil, and his support for logging and mining projects that continue as key industries in the Amazon, personify the relentless drive for accumulation that trumps human and ecological reproduction.

The manner of the defeat of the Workers’ Party and the extent to which its industrial base was hollowed out through co-option, and demoralised by austerity and corruption scandals has left organised labour fragmented and disoriented. The inability of CUT to organise effective general strikes during the impeachment of Dilma and the jailing of Lula was palpable. In the absence of coordinated struggle in the cities, the most visible resistance to this contemporary phase of authoritative capitalism is laid bare in the assassination of land activists in the Amazonian region defending territories coveted by illegal loggers and multinationals alike. Recent online strikes by platform workers and the escalating street protests against Bolsonaro illustrate the fermenting of an opposition to the Bolsonaro regime. These are yet to be forged into coherent alliances or strategies, yet serve as a reminder that neither elitist authoritarianism nor creative forms of resistance across urban and rural abodes are an exception in Brazil’s history.

## Notes

1. This first section updates the information provided by Garvey and Torres for the Conversation article, ‘Brazil’s Jair Bolsonaro is devastating indigenous lands, with the world distracted’, 30 May 2020.

2. ‘The Bolsa Família Program (PBF) is a federal program for the direct transfer of income to families in poverty or extreme situations, with the purpose of promoting their access to basic social rights and breaking with the intergenerational cycle of poverty. The Program is [. . .] linked to the fulfillment of commitments [conditionalities] in Health, Education and Social Assistance. [. . .] to ensure the provision of basic actions, and to enhance the improvement of the quality of life of families and contribute to their social inclusion. The pbf health agenda in the SUS includes the provision of services for the performance of prenatal care by pregnant women, the monitoring of child growth and development and immunization of children. [. . .]” (Ministry of Health). The Bolsa Familia was a development of the Bolsa Escola (2001–2003), which was a monetary payment to women conditional on their children’s regular school attendance from age 6–15 years.

3. The impact on many households was certainly demonstrable: the closure of the inequality gap (along with Bolivia, Venezuela and Ecuador at this time), something like a tripling of Black students in university; countless new universities and so on.

4. See Antunes (2019).
